# Possible Long Term Effects of Chemical Warfare Using Visual Evoked Potentials

**Published:** 2014-09

**Authors:** Abbas Riazi, Rhamatollah Hafezi, Mahmoud Babaei, Mostafa Naderi

**Affiliations:** 1 Department of Ophthalmology, School of Medicine, Baqiyatallah University of Medical Sciences, Tehran, Iran;; 2 Department of Physical Medicine and Rehabilitation, School of Medicine, Baqiyatallah University of Medical Sciences, Tehran, Iran

**Keywords:** Chemical warfare, Visual evoked potential, Long term effects

## Abstract

Some studies have already addressed the effects of occupational organic solvent exposure on the visually evoked potentials (VEPs). Visual system is an important target for Sulphur Mustard (SM) toxicity. A number of Iranian victims of Sulphur Mustard (SM) agent were apprehensive about the delay effect of SM on their vision and a possible delay effect of SM on their visual cortex. This investigation was performed on 34 individuals with a history of chemical exposure and a control group of 15 normal people. The Toennies electro-diagnosis device was used and its signals were saved as the latencies. The mean of N75, N140 and P100 of victims of chemical warfare (VCWs) and control group indicated no significant results (P>0.05). The VCWs did not show any visual symptoms and there was no clear deficit in their VEPs.

## Introduction

Visual system is one of the main targets for organic solvents toxicity.^1^ Contrast sensitivity reduction in the intermediate special frequency has been reported as a consequent of this exposure. Colour vision is an indicator of the toxicity of several solvents and chemicals.^2^ Prenatal solvent exposure is associated with selective visual deficits, including reduced contrast sensitivity and abnormal red–green discrimination.^3^

Sulphur mustard (SM) was the most widely used chemical warfare agent (CWA) during the Iran–Iraq war (1983-1988), resulting in chemical casualties.^4^ Ocular complications of SM includes early manifestations of corneal penetration in the first minutes, conjunctival necrosis and vascular occlusion during the first 24 hours and corneal vascularisation as well as ulceration (perforation) within a few minutes of severe exposure.^5^ VCWs have shown a number of delayed ocular complications.^6^ The long-term effects of some chemicals like sarin were reported as EEG abnormalities beyond one year after exposure,^7^ abnormal evoked potentials latencies (P300 and VEP-P100) and vestibulocerebellar damage.^8^ VEP is commonly used to detect abnormality of visual pathway in patients having vision loss without any ocular manifestations. VEPs studies^9^ can be used in experimental toxicology investigations.^10^ There are many reports indicating the effects of organic solvent and other toxic chemicals on the VEPs.^11^^,^^12^ Measuring VEP latency is more common than VEP ampliude.^13^^,^^14^ Since a number of VCWs were concerned about their vision, this study was instigated to determine possible long term effect on their visual pathways and cortex.

## Patients and Methods

VCWs with a history of SM exposure with differing severities of eye injuries (normal, mild, moderate) were recruited for this study. An age-matched control group was also assembled for comparison. Participants with systemic diseases such as diabetes as well as disorders other than exposure to SM were excluded from data analysis. VCWs that had severe and very severe eye lesion were excluded. Confirmation of SM exposure leading to chemical injury was confirmed from the medical records of the Chemical Casualty Registry of the Veterans Foundation. The Toennies electro-diagnosis device was used for the measurement of VEPs and the latencies of the Flash VEP (FVEP) components (N75, P100 and N140) were assessed. The monitor was placed at 1.5 m with 100% contrast sensitivity and 5 cycles per degree. Electrodes were less than 2KΩ with 1Hz pulse excitation. VEP Electrodes were placed according to the International 10/20 system. The locations of electrodes were determined according to bony landmarks and additional electrodes were attached to the forehead and ear lobes. The signals were saved as N75, P100 and N140. The VEP signals were recorded in two rounds in order to access recording accuracy. The results were analysed using SPSS software and unpaired student t-testing was used to compare the groups. 

This study was carried out in 2005 at Baqiyatallah Hospital (Tehran, Iran) and was approved by the Ethics Committee of Baqiyatallah University of Medical Sciences. All participants were debriefed on the project and its procedure in their native language followed by obtaining verbal agreement for the eye examinations.

## Results

This study was performed on 35 VCWs (68 eyes, two subjects were monocular) and a control group of 15 normal people (30 eyes). The mean age of VCWs and normal participants was 42.15±10.72 (SD) and 41.68±4.81 (SD) years respectively. Among the VCWs, there were 16 eyes with normal vision and 40 with some degree of refractive errors which were correctable to normal visual acuity with spectacle lenses. Although there were 4 eyes with abnormal visual acuity (>20/20, >20/30) even with corrective lenses, their VEPs results were normal. Majority of patients had normal corneas. However, 3 eyes had moderate corneal opacity and the VEPs results were abnormal. Additional abnormal VEPs result belonged to a patient with a piece of metal in his brain as well as a patient with diabetic. In the VCWs group there were two monocular individuals for which due to enucleation, their VEPs results were normal. The control group had no history of poisoning and SM contamination. Among the 32 eyes, 26 had normal vision and the rest had some degree of refractive errors which had been corrected by spectacle. The VEPs results among the control group were within the normal limits. 

In the first round, the mean of N75, N140 and P100 of VCWs and control indicated no significant results (P>0.05). In the second round, there was a significant difference of N140 latency between the VCWs and controls. However, there was no significant difference of N75 and P100 in both group (P>0.05). In both rounds, the mean of N75, N140 and P100 indicated no significant results (P>0.05) for both right and left eyes of VCWs. Table 1 and 2 presents detail statistical results.

**Table 1:** N75, P100 and N140 latencies in affected and normal control subjects. Both testing occasions

		**VCW **		**Normal**		
**Feature**	**Round of testing**	** mean latency**	**σ **	**mean latency **	**σ **	**Difference**
N75	1	76.7	8.1	76.5	5.0	ns
	2	76.7	8.5	76.5	5.7	ns
P100	1	104.7	8.0	104.0	8.5	ns
	2	104.8	8.5	105.1	4.28	ns
N140	1	137.6	6.8	138.5	6.8	ns
	2	138.5	6.8	135.5	7.0	s

**Table 2:** Comparison of right and left N75, P100 and N140 latencies in affected subjects, both testing occasion

		**Right Eye **		**Left Eye**		
**Feature**	**Round of testing**	** mean latency**	**σ**	**mean latency**	**σ **	**Difference**
N75	1	76.7	8.1	76.5	5.0	ns
	2	77.8	4.22	76.6	5.8	ns
P100	1	102.8	7.0	106.5	8.8	ns
	2	103.9	3.8	105.6	4,7	ns
N140	1	136.2	6.2	139.1	7.0	ns
	2	134.3	8.4	136.0	5.5	ns

## Discussion

VEP alone cannot be considered as a comprehensive eye examination. Abnormal VEP may also be attained in individuals with poor fixation and conscious suppression. This study showed minimal long term effect of SM. During the second round of recording, a significant difference in the latency of N140 between the two groups was observed. Although this finding is statistically significant, it occurred only during the second round and only in N140. Thus, these changes should be seen in all dimensions on VEPs. Since the subjects had only concerns about their vision and had no significant visual disturbances, its interoperation as a long term effects of SM on visual cortex is debatable.

The majority of VCWs still suffer from the long-term effects of SM even after 20 years of contamination.^15^ Visual disturbance is one of the main concerns regarding such delayed effects of SM among VCWs. In recent years, the situation of refractive errors has been investigated to discover delay effects.^6^ This study was instigated due to the lack of information on of the effects of SM on the visual pathway. The finding from VEPs as a long term effects, revealed no significant abnormality in the visual pathway.

There were 40 VCWs eyes with some degree of refractive errors which were correctable to normal visual acuity. However, there were four eyes with poor vision even with corrective lenses, but their VEPs results were normal. Consequently, vision problem among VCWs cannot be considered as a long term effect of SM on their VEPs. Furthermore, majority of VCWs had normal corneas. However, three eyes had moderate corneal opacity and their VEPs were abnormal. It should be noted that a few abnormal records of VEPs in the VCWs were mainly in relation to either ocular pathology (e.g. corneal opacity) or systemic diseases (e.g. diabetes). In one case, the abnormality in the VEPs was in relation to very small pieces of metal lodged in the occipital lobe of the brain. 

Limitation: insufficient number of subjects (VCWs) was a limitation of this study. 

## Conclusion

This study did not reveal abnormal VEPs in VCWs. It is concluded that there is insignificant long-term effects of SM on visual system.
